# Medical education in times of COVID-19: survey on teachers' perspectives from a German medical faculty

**DOI:** 10.3205/zma001489

**Published:** 2021-06-15

**Authors:** Anne Herrmann-Werner, Rebecca Erschens, Stephan Zipfel, Teresa Loda

**Affiliations:** 1 University Hospital Tuebingen, Department of Internal Medicine VI/Psychosomatic Medicine and Psychotherapy, Tuebingen, Germany; 2 Eberhard-Karls University of Tuebingen, Faculty of Medicine, Tuebingen, Germany

**Keywords:** medical education, COVID-19, teacher, mental well-being, digitalization

## Abstract

**Background: **Clinicians in their role as teachers and medical faculties were struggling to address the medical students’ needs regarding their medical education in times of COVID-19. They were especially confronted with several challenges regarding what medical training should look like and how to transfer medical training to integrate relevant skills like interpersonal or practical competencies. This study aimed to investigate the teachers’ perspectives on medical education and COVID-19 in general, including their distress level.

**Methods: **This quantitative questionnaire study was distributed online among responsible lecturers of medical training at the Medical Faculty of Tuebingen. Teachers answered questions about the medical training, COVID-19 in general (on a seven point- Likert scale from “not at all” to “completely”) and their mental well-being (STAI). Descriptive data analysis and t-tests were performed.

**Results:** The teachers reported being significantly (p<.01) more distressed regarding the medical training (*M*=4.63, *SD*=1.24) in comparison to their private lives (*M*=3.58, *SD*=1.38) or the clinical context (*M*=3.33, *SD*=1.95). They also felt significantly less informed about the medical training in times of COVID-19 (p<.001). They wished for more support and information from their medical faculty. When teachers were asked which teaching should be implemented in future, they reported the most the online lectures (87.5%), followed by collaborative working (75.5%), live broadcast (62.5%) and online chats (58.3%). Teachers also saw the current situation of COVID-19 as a chance for a digital transformation of the medical education (*M*=5.92, *SD*=0.95).

**Discussion: **Teachers of the Medical Faculty of Tuebingen saw online-based teaching formats as a chance to meet the medical students’ needs regarding the medical education. Video-based formats like online lecturers and online chats with teachers might play a relevant role in order to impart knowledge. Furthermore, medical students should also be taught in digital formats like telehealth, including patient-physician distance interactions.

## Zusammenfassung

**Hintergrund: **Dozierende Klinikärzte/-ärztinnen und medizinische Fakultäten hatten es schwer, den medizinischen Ausbildungsbedürfnissen der Medizinstudierenden in Zeiten von COVID-19 gerecht zu werden. Insbesondere waren sie vor die Herausforderung gestellt, wie die medizinische Ausbildung unter den gegebenen Bedingungen aussehen könnte und wie sich die Vermittlung maßgeblicher zwischenmenschlicher oder praktischer Fähigkeiten integrieren ließe. Diese Studie hatte zum Ziel, die Perspektiven der Dozierenden hinsichtlich der medizinischen Ausbildung und COVID-19 im Allgemeinen zu untersuchen, einschließlich ihres eigenen Stresslevels.

**Methoden:** Die Fragebögen dieser quantitativen Erhebung wurden den Dozierenden der Medizinischen Fakultät Tübingen online zur Verfügung gestellt. Die Dozierenden beantworteten Fragen zur medizinischen Ausbildung, zu COVID-19 im Allgemeinen (anhand einer 7-stufigen Likert-Skala von "überhaupt nicht" bis "sehr") und zu ihrem psychischen Wohlergehen (STAI). Es wurden deskriptive Datenanalysen und and t-Tests durchgeführt.

**Ergebnisse: **Die Dozierenden gaben an, dass sie sich in ihrer medizinischen Lehrtätigkeit signifikant mehr (p<0,01) belastet fühlten (*M*=4,63, *SD*=1,24) als in ihrem Privatleben (*M*=3,58, *SD*=1,38) oder ihrer klinischen Tätigkeit (*M*=3,33, *SD*=1,95). Ferner fühlten sie sich hinsichtlich ihrer Lehraufgabe unter COVID-19-Bedingungen signifikant weniger informiert (p<0,001). Sie wünschten sich mehr Unterstützung und Informationen von ihrer medizinischen Fakultät. Auf die Frage, welche Lehrformen in Zukunft implementiert werden sollten, gaben die Dozierenden überwiegend Online-Vorlesungen an (87,5%), gefolgt von kollaborativem Arbeiten (75,5%), Live-Übertragungen (62,5%) und Online-Chats (58,3%). Die Dozierenden sahen die aktuelle Situation mit COVID-19 auch als Chance für eine digitale Transformation der medizinischen Ausbildung an (*M*=5,92, *SD*=0,95).

**Diskussion: **Dozierende der Medizinischen Fakultät Tübingen sahen Online-Lehrformate als Chance an, um den Bedürfnissen der Medizinstudierenden in ihrer medizinischen Ausbildung gerecht zu werden. Videobasierte Formate wie Online-Vorlesungen und Online-Chats mit Dozierenden könnten bei der Wissensvermittlung eine wichtige Rolle spielen. Darüber hinaus sollten Medizinstudierende auch in digitalen Formaten wie der Telemedizin ausgebildet werden, einschließlich der Fern-Interaktion zwischen Arzt/Ärztin und Patient/-in.

## 1. Introduction

The Coronavirus pandemic challenged medical education worldwide [1]. The start of the summer term 2020 was delayed at German medical faculties, and clinical clerkships and important exams were suddenly cancelled in order to minimise personal contact to mitigate the spread of COVID-19 [2]. The Association of American Medical Colleges even suggested in guidelines that medical schools should pause clinical rotations for medical students [https://www.aamc.org/]. 

Though the medical training was stopped at German medical faculties, medical students mobilised and were utilised to support the health care system in the combat against COVID-19 [3], [https://www.bvmd.de/]. Regarding this support of medical students, physicians were especially challenged in their role as clinicians as well as teachers in times of COVID-19. Since keeping a distance is the current way to fight the pandemic of COVID-19, questions arose regarding what teaching could look like and how to ensure medical students’ progress forward or graduation. 

The medical training had to be transferred into a digital format, though it has traditionally been structured as in-person learning [1]. Medical teachers needed to be innovative and creative to maintain the quality of medical training by combining technology-enhanced learning experiences with traditional ones [4]. Online video-conferencing platforms were used to implement lectures and small-group learning. However, Newman (2020) argued that these online teaching formats might comprise only a minor component of the previously existing curriculum before COVID-19 [5]. Sandhu & Wolf (2020) emphasised that the online formats need to be adapted to deliver teaching on clinical and practical skills that would have otherwise been developed during in-person courses. 

In the UK, final-year medical students were dramatically affected by COVID-19, as their medical training stopped, and there was discussion regarding whether they should fast-track through their studies independently if they felt able to take on this responsibility [1], [6]. Core competencies for medical doctors, like communication or interpersonal skills, must be integrated into online teaching formats to ensure the development of empathic behaviour in patient-physician encounters [7], [https://www.acgme.org/Newsroom/Blog/Details/ArticleID/10281/ACGME-e-Communication-May-26-2020]. 

Previous literature focussing on COVID-19 and medical education offers a broad variety of suggestions when recommending digital teaching possibilities, e.g. implementing virtual reality settings [2], [8]. In particular, they all agreed on the point that medical training needs a digital transformation in general and that single interventions won’t be enough [1], [9], [10]. 

Therefore, the following questions arose regarding medical training in times of COVID-19:

- What should medical training look like in times of COVID-19?
- How to transfer classes – particularly teaching interpersonal and practical skills – best into online teaching?

### 1.1. Aim of the study

What did the medical training look like in its practical implementation in summer term 2020, and how did the teachers experience this challenge? 

This survey aimed to investigate the medical training, including stressors and expectations from the perspectives of teachers at one medical faculty in Germany during the 2020 summer term. Furthermore, we asked them for their mental well-being and their attitude concerning COVID-19. 

## 2. Materials and methods

### 2.1. Study design, participants and procedure 

This survey presents a quantitative study that was performed at the Medical Faculty of Tuebingen. The assistant lecturers of this faculty were invited via email to participate in the online survey, which started in May 2020 and lasted four weeks. 

#### 2.2. Ethics 

The survey was approved by the Ethics Committee of Tuebingen Medical Faculty (no. 314/2020BO2). Participation was voluntary, and the data acquisition was kept anonymous. All participants gave their written consent. 

#### 2.3. Measurements

##### 2.3.1. Demographic variables 

Demographic data like age and gender were assessed. Furthermore, teachers were asked for their teaching experience and their qualifications. 

##### 2.3.2. Topics concerning COVID-19 

Teachers were asked about their current distress level in times of COVID-19 regarding issues of their private lives, clinical work and teaching. Furthermore, they rated their level of knowledge about COVID-19 in general, as well as in the medical and teaching contexts. All questions were rated on a seven-point Likert scale ranging from 1 (“not at all”) to 7 (“completely”). Last, they reported if they had COVID-19, how much they were afraid of it and what they estimate as their risk of getting infected with COVID-19 (0% to 100%). 

##### 2.3.3. Teaching

Teachers rated their desired and expected course content for the summer semester 2020 as well as possible long-term changes in their medical education due to the circumstances of the pandemic (i.e. multiple-option items). Furthermore, they ranked their expectations to students in this summer term. Using a seven-point Likert scale from 1 (“not at all”) to 7 (“completely”), they rated how much the COVID-19 crisis presented a chance for a digital transformation in the medical faculties. In open questions, participants reported their stressors regarding medical education in the COVID-19 pandemic and what they expected from their medical faculty. They could also add comments regarding medical education in times of COVID-19. 

##### 2.3.4. Mental well-being measurements

For measurement of mental well-being, the State-Trait Anxiety Inventory (STAI) [11] questionnaire was used to assess the teachers’ distress levels, particularly, the “state anxiety” dimension was used to measure the degree to which they felt distressed, using a four-point scale, from 1 (“not at all”) to 4 (“very much so”). They also rated their wish for recommendations on how to cope with distress, for relaxation techniques or psychotherapy. Additionally, they rated their ability to handle a crisis in general. These questions were rated on a seven-point Likert scale, ranging from 1 (“not at all”) to 7 (“completely”).

#### 2.4. Data analysis 

Data were normally distributed as tested by the Kolmogorov-Smirnov test. Descriptive data like mean values (M), standard deviations (SD), sum scores, frequencies and percentages of relevant factors were calculated. Missing data were replaced by mean. In order to compare the results, independent samples t-test and Pearson correlations were conducted. The level of significance was p<.05. IBM SPSS Statistics version 26 was used for data analysis.

## 3. Results

### 3.1. Demographics 

Twenty-four out of 42 teachers finished the survey (response rate=57.1%). Mean age of the teachers was 44.8 (*SD*=8.8). Eight participants were female (33.3%), and 16 were male (66.7%). The average teaching experience was 15.7 years (*SD*=8.7), ranging from three to 40 years. Eighteen teachers (75.0%) reported having a specific qualification in medical education. Please see attachment 1 for more details. 

#### 3.2. Topics concerning COVID-19 

Teachers reported being distressed regarding issues in their private lives with 3.6 (*SD*=1.4) and clinical work with 3.3 (*SD*=2.0). However, they were significantly more distressed with 4.6 (*SD*=1.2) regarding medical education, (*t*(45)=-2.77, *p*<.01). Their wishes for psychotherapy and relaxation techniques were low with 2.0 (*SD*=1.1) for psychotherapy and 2.1 (*SD*=1.3) for relaxation techniques. Their wishes for recommendations on how to cope with distress were higher with 2.6 (*SD*=1.4), but this difference was not significant. 

When regarding their current level of information, they felt well informed about COVID-19 in general with 6.0 (*SD*=1.0) and in the clinical context with 5.9 (*SD*=1.0). However, they felt significantly less informed with 3.9 (*SD*=1.4) regarding the medical training in times of COVID-19 (*t*(41)=6.29, *p*<.001).

Twenty-one (87.5%) reported not having been infected with COVID-19, and two participants (8.33%) did not provide any information. The fear of getting infected was 17.1%, and the participants estimated the probability of getting infected at 34.6%.

Teachers reported the following stressors triggered by COVID-19: lack of information, lack of interaction with students, transfer to online teaching, double burden by combining clinical work and teaching, and home office without childcare. 

#### 3.3. Teaching 

Teachers rated desired and expected content for the COVID-19 summer semester as well as possible long-term changes in medical education. Please see table 1 (Tab. 1) for results. 

**Figure 1:**
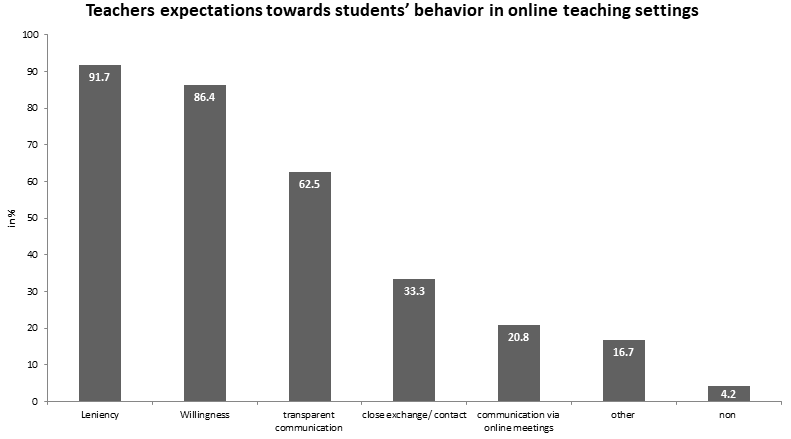
Teachers expectations towards students’ behavior in online teaching settings

Furthermore, they reported seeing the COVID-19 crisis as a chance for a digital transformation in medical training, with 5.9 (*SD*=1.4). They also expected the medical faculty to show more transparency, make clearer announcements and be more open in dialogue. They also wished for licences for rugged video-based conferences and for more support regarding the digitalisation. In addition, they ranked their expectations to the students. Please see figure 1 (Fig. 1) for more details. 

#### 3.4. Mental well-being 

The current distress level of the teachers was high when regarding the STAI (cut-off >43), with 44.3 (*SD*=5.9). Simultaneously, they reported being able to handle the crisis, with 5.3 (*SD*=1.0). 

## 4. Discussion

This survey aimed to assess teachers’ perspectives on medical training and their current distress levels in times of COVID-19. With regard to the content of medical education during the COVID-19 pandemic, teachers ranked online lectures as highest, followed by collaborative working, live broadcast, and online chats. Teachers reported being significantly more distressed about medical education compared to issues in their private lives or clinical work. However, they generally reported being able to cope with the distress. 

### 4.1. Teachers’ perspectives on COVID-19 and medical education

Participating teachers reported being highly distressed regarding providing medical training, but felt less distressed in their private lives about COVID-19 in general. These high levels of distress represented a fact several authors have already addressed: teachers have needed to adapt their teaching formats in a very short period of time while simultaneously maintaining a high quality of medical training [2], [5], [8]. Thus, teachers were especially challenged. Additionally, they wished for more information on how COVID-19 might impact their medical training in the summer term. They expected faculty members to have more transparency on the topic. They also wished for more support in transferring their teaching to a digital format, mirroring the general notion of unpreparedness in use of modern technologies for teaching. 

Similar to other studies, the teachers in this study expected that traditional teaching formats like lectures would easily transfer into digital formats, like online lectures [1], [8], [9], [12]. Moreover, they saw independent collaboration as an important method to impart knowledge in times where in-person contact was not possible [2]. Thus, teachers sought to implement online chats with their students to keep personal contact despite social distance. In general, the results showed that teachers considered the COVID-19 crisis as a potent opportunity for a digital transformation of medical training. Medical students also needed to be trained in telehealth, including technological aspects and professional models of physician-patient distance interactions [13]. Moreover, teachers might have to support medical students in finding their role in the medical community, as it will likely be challenging to evolve and integrate themselves due to missed clinical rotations and collaborative experiences in times of COVID-19 [2]. 

This optimistic attitude of teachers involved some expectations of medical students in order to successfully implement online teaching modules in the summer term. They expected medical students to be lenient and patient and to demonstrate that they are prepared for online medical education. 

Loda et al. (2020) also questioned students regarding their expectations of medical training under COVID-19 [14]. Similar to teachers, students felt less informed about their medical training in times of COVID-19 compared to private or general information. Additionally, they also felt stressed by their medical studies. However, they were also optimistic that their teachers will enhance their digital competencies during the pandemic. 

Moreover, teachers were challenged in their role as clinicians. They reported on the double burden of combining teaching with clinical work, which was very stressful for them. Future potential stressors were lack of information from the government and their bosses as well as working at home without childcare. Interestingly, teachers did not express a need for relaxation techniques or psychotherapy to manage their stress. This may be due to different concepts and understandings of terms. 

#### 4.2. Implications 

Based on the results, some implications may be gleaned. Medical faculties should strive to improve communication in a clear and transparent way so that teachers feel sufficiently informed. Furthermore, training on how to conduct online teaching courses should be offered to teachers. Such trainings would help teachers to learn how to use digital learning platforms or video conference systems as well as the various features (such as interactive whiteboards) that these tools have. Simultaneously, medical students should also be trained in order to establish clear communication with their teachers in online courses. Such training will very likely help teachers and students transfer their medical education into the digital world. Most online experiences in response to the COVID-19 pandemic are not sufficiently adaptable to medical school training and need to be improved for future online teaching [15]. 

#### 4.3. Limitations 

We are aware that our results are limited as they represent teachers from only one medical facility, and the numbers of participants was limited to 24. However, we think that it might be of interest to explore how medical training could be implemented in times of COVID-19. With regard to data analysis, we need to consider that the mean imputation might lead to a bias of variance. However, the overall mean of missing values was estimated at 1.28%, which is very low. 

## 5. Conclusion

This survey aimed to assess teachers’ perspectives on medical education during the COVID-19 pandemic. In line with previous literature, participating teachers saw a need for medical schools to continue classes, and new ways of teaching were developed through online-based learning platforms that facilitate online lecturers, live broadcasts, and online chats with students [1], [5], [8]. The results of this study present a practical implementation of various teaching formats in medical education in times of COVID-19. Furthermore, stressors, expectations, and the mental well-being of teachers in this unique situation due to COVID-19 were assessed showing an additional stressor with regards to teaching but generally a feeling of good situational coping.

## Authors’ contributions

AHW and TL were responsible for the design and conduction the study, as well as acquisition, analysis and interpretation of data. AHW and TL drafted the first version of the manuscript. RE was involved in data analyses and interpretation and revised the manuscript critically. SZ made substantial contributions to the study design and revised the manuscript critically. All authors approved the final version of the manuscript and agreed to be accountable for all aspects of the work. 

## Declarations

### Ethics approval and consent to participate 

Ethical approval for the study had been given by the Ethics Committee of Tuebingen’s Medical Faculty. Students signed their consent to participate. 

#### Availability of data and material 

The datasets used and/or analysed during this study are fully available without restriction. 

## Acknowledgements

We would like to thank Lea Herrschbach, B.Sc. for her study assistance. We acknowledge support by Deutsche Forschungsgemeinschaft and Open Access Publishing Fund of University of Tuebingen.

## Competing interests

The authors declare that they have no competing interests. 

## Supplementary Material

Numbers of teachers’ specialty

## Figures and Tables

**Table 1:**
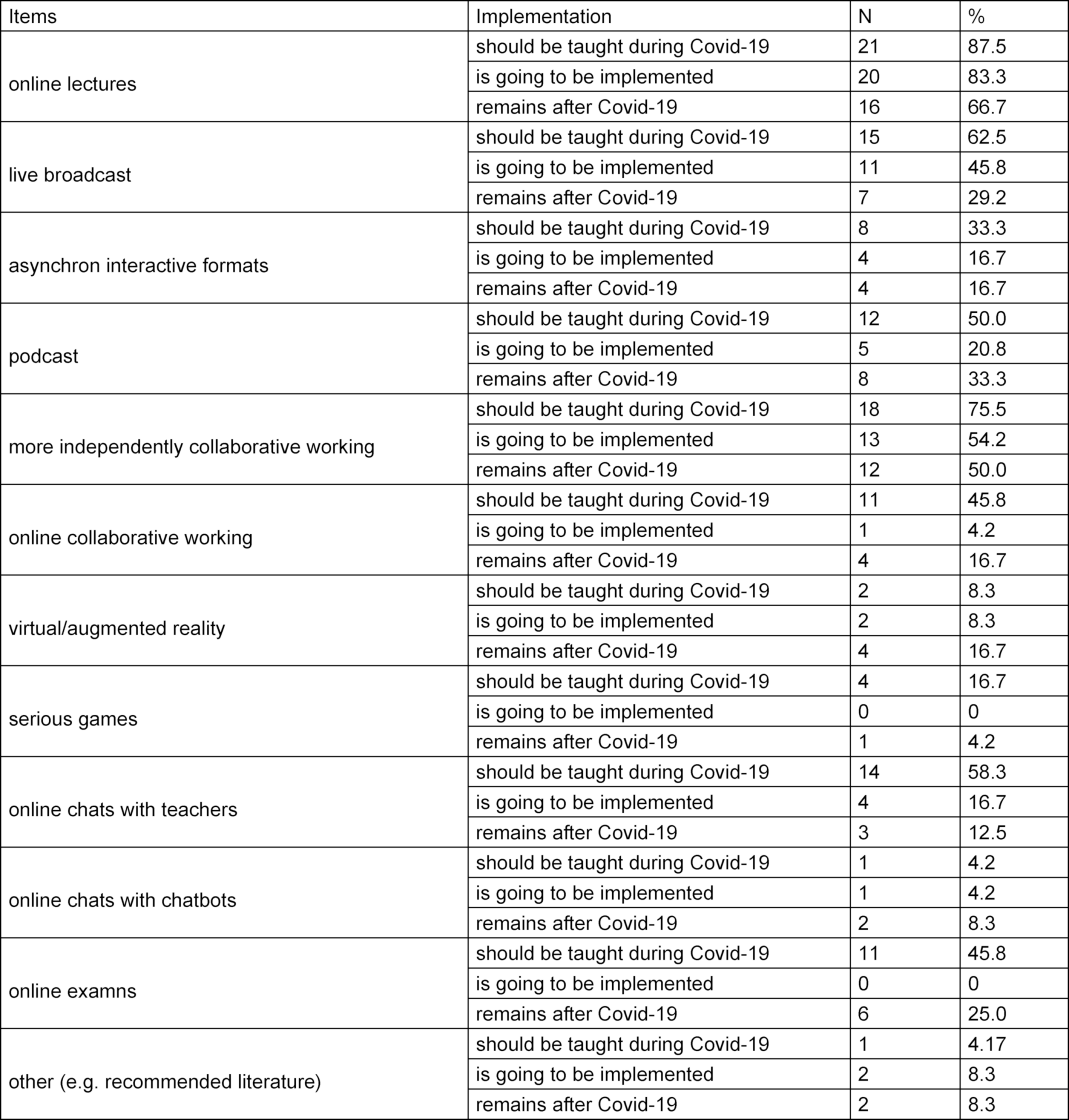
Results
