# Supplementary material for: Medical education in times of COVID-19: survey on teachers' perspectives from a German medical faculty
Source: GMS J Med Educ. 2021 Jun 15;38(5):Doc93. doi: 10.3205/zma001489 (PMC8256125; doi:10.3205/zma001489)
Supplement: Numbers of teachers’ specialty [file JME-38-5-93-s-001.pdf]

Attachment 1: Numbers of teachers' specialty

specialty	N	%[1]
Anatomy	1	4.2
Ophthalmology	1	4.2
Surgery	1	4.2
Dermatology	1	4.2
Human genetics	3	12.5
Human Medicine	1	4.2
Internal Medicine	4	16.7
Paediatrics	1	4.2
Medicine	1	4.2
Medical Psychology and Sociology	1	4.2
Microbiology and Hygiene	1	4.2
Neurology	1	4.2
Oncogenetics	1	4.2
Plastic and reconstructive medicine	1	4.2
Psychosomatics	2	8.3
Radiology	1	4.2
Trauma surgery	1	4.2
Missing	1	4.2
Total	24	100.0

[1] Percentages are rounded to one decimal.

Attachment 1 to: Herrmann-Werner A, Erschens R, Zipfel S, Loda T. *Medical education in times of COVID-19: survery on teachers' perspectives from am German medical faculty.* GMS J Med Educ. 2021;38(5):Doc93. DOI: 10.3205/zma001489
